# Case report: hepatic arterial infusion chemotherapy combined with sintilimab and lenvatinib for conversion therapy of colorectal cancer liver metastases

**DOI:** 10.3389/fimmu.2023.1325445

**Published:** 2023-12-15

**Authors:** Kai Peng, Yuhua Li, Hao Su, Chenlu Lan, Zaida Huang, Yongguang Wei, Xiwen Liao, Minhao Peng, Tao Peng, Guangzhi Zhu

**Affiliations:** ^1^ Department of Hepatobiliary Surgery, The First Affiliated Hospital of Guangxi Medical University, Nanning, China; ^2^ Guangxi Key Laboratory of Enhanced Recovery After Surgery for Gastrointestinal Cancer, Nanning, China; ^3^ Key Laboratory of Early Prevention & Treatment for Regional High Frequency Tumor (Guangxi Medical University), Ministry of Education, Nanning, China

**Keywords:** hepatic arterial infusion chemotherapy, colorectal cancer liver metastasis, conversion therapy, interventional therapy, case report

## Abstract

**Background:**

Liver metastasis is one of the most common causes of death in patients with colorectal cancer. Therefore, improving the treatment effect of liver metastatic carcinoma of colorectal cancer is also one of the effective ways to improve the survival time of patients with colorectal cancer. The main treatment method for liver metastasis of colorectal cancer is preoperative neoadjuvant chemotherapy through intravenous administration. However, no one has reported a conversion therapy approach for the treatment of colorectal cancer liver metastases patients through arterial infusion chemotherapy combined with targeted agents and PD-1 monoclonal antibody. This case reports a conversion therapy method of liver metastases of colorectal cancer by hepatic arterial infusion chemotherapy (HAIC), sintilimab injection combined with lenvatinib to achieve radical resection of liver metastatic carcinoma after treatment.

**Case presentation:**

The patient was a 69-year-old man who had previously undergone laparoscopic left hemicolectomy for descending colorectal cancer and multiple interventional and surgical treatments for hepatocellular carcinoma. During this treatment, the patient underwent radiological and serological tests, and primary liver cancer was considered at the initial diagnosis stage. Therefore, this liver malignant tumor lesion was treated according to the primary liver cancer treatment protocol before surgical resection. Therefore, the patient received HAIC combined with sintilimab injection and lenvatinib. After three treatment cycles, radiological examination showed no obvious tumor activity, alpha-fetoprotein (AFP) decreased to normal, protein induced by vitamin K absence or antagonist II (PIVKA II) decreased significantly, and the curative effect was evaluated as complete remission. Subsequently, we performed surgical resection of this liver lesion. The pathological response of left lobe tumor was partial remission (PR). Most of the tumors were necrotic and the necrosis rate was greater than 95%. A small amount of live tumor tissue remains (<5%). The pathological classification of this tumor was confirmed as moderately differentiated adenocarcinoma by immunohistochemical staining of multiple tumor indicators in the pathology department. No significant adverse drug events were observed in this patient during treatment.

**Conclusion:**

Hepatic arterial infusion chemotherapy combined with sintilimab injection and lenvatinib conversion therapy provides the opportunity for radical surgical resection of colorectal cancer liver metastases.

## Introduction

Liver is the most important target organ for hematogenous metastasis of colorectal cancer, and liver metastasis of colorectal cancer is one of the key and difficult points in the treatment of colorectal cancer (1). A total of 15%–25% of patients with colorectal cancer will have liver metastases at diagnosis, while another 15%–25% will have liver metastases after radical resection of the primary colorectal cancer, of which 80%–90% of liver metastases cannot be radically resected initially (2–4). Liver metastasis is also the leading cause of death in patients with colorectal cancer (5). The median survival of patients with untreated liver metastasis is only 6.9 months, and the 5-year survival rate of patients with unresectable liver metastases is less than 5% (6, 7), while the median survival rate of patients with liver metastases that can be completely resected is 5 months, and the 5-year survival rate can reach 30%–57% (8, 9).

For patients with a previously radical colorectal primary resection and no recurrence of the primary lesion, the liver metastases can be completely resected and the liver is less than 70% resected (without cirrhosis), the liver metastases should be surgically resected, or neoadjuvant therapy can be performed first (1).

Neoadjuvant therapy can be used in patients with primary colorectal cancer who have undergone radical resection and have not received chemotherapy, or in patients with liver metastasis who have undergone radical resection and postoperative replacement 12 months before liver metastasis; that is, neoadjuvant chemotherapy and concurrent chemotherapy before surgery can achieve a higher complete response rate, which is conducive to organ preservation, can reduce the occurrence of distant metastasis, and can improve long-term survival. Conventional systematic chemotherapy regimens include FOLFOX, FOLFIRI, CapeOX, or FOLFOXIRI. There is still controversy over whether to use molecular targeted therapy in combination, and hepatic arterial infusion chemotherapy (HAIC) may also be considered. For patients who received chemotherapy within 12 months before the discovery of liver metastases, it is generally believed that the effect of neoadjuvant chemotherapy may be limited, and the liver metastases should be directly resected and postoperative adjuvant therapy (10) or neoadjuvant therapy should be considered after changing chemotherapy regimen (11, 12), or preoperative combined with hepatic artery infusion chemotherapy (13). Cytotoxic T lymphocyte-associated protein 4 antibody and programmed death receptor 1 antibody are representative agents of immune checkpoint tumor suppression, which can enhance the activity of immune cells to kill tumor cells, and are widely used in tumor immunotherapy (14, 15). Sintilimab injection is a PD-1 inhibitor. Multi-target tyrosine kinase inhibitors not only target factors that promote tumor angiogenesis, but also inhibit a variety of kinases related to tumorigenesis. Lenvatinib not only inhibits the kinase activity of vascular endothelial growth factor receptor-3 (VEGFR1-3), but also inhibits fibroblast receptor (FGF) and its receptors FGFR1–4, PDGFRα, and stem cell factor receptor, thereby inhibiting tumor vessels (16). It has been reported that sequential target immunotherapy can enhance the killing effect of immune cells on tumor. This case reports the efficacy of local HAIC combined with sintilimab injection and lenvatinib in the treatment of colorectal cancer liver metastases.

## Case presentation

**Figure 1:**
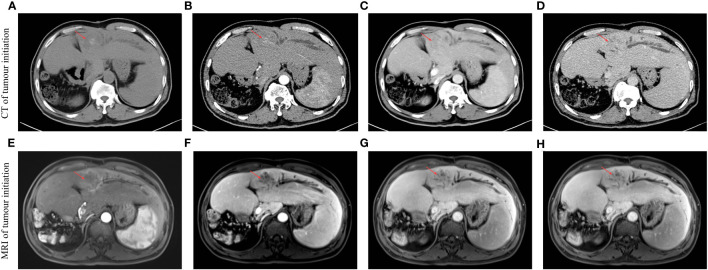
Multistage liver-enhanced CT and gadoxetate disodium-enhanced MRI scans of the liver showed that S3 and S4 occupied the liver at the first visit. The red arrow indicates an intrahepatic tumor. **(A)** Initiation-CT image in the period of non-contrast scan. **(B)** Initiation-CT image in the period of arterial phase. **(C)** Initiation-CT image in the period of portal vein phase. **(D)** Initiation-CT image in the period of delay phase. **(E)** Initiation-MRI image in the period of arterial phase. **(F)** Initiation-MRI image in the period of portal vein phase. **(G)** Initiation-MRI image in the period of non-contrast scan. **(H)** Initiation-MRI image in the period of hepatobiliary phase.

This study was approved by the Ethics Committee of The First Affiliated Hospital of Guangxi Medical University (the approval number is 2023-E637-01). A 69-year-old male patient was admitted to the hospital 10 days after reexamination for hepatic space-occupying lesions on 17 March 2022. He had a history of diabetes, clonorchiasis sinensis, and gout. He had been treated with deinfestation, and his blood glucose and uric acid control was satisfactory. Assessment and examination were performed after admission. Liver CT and liver MRI indicated that S3 and S4 occupied liver space (**Figures 1A–H**), and tumor recurrence was considered. Alpha-fetoprotein (AFP): 716.27 ng/mL, protein induced by vitamin K absence or antagonist II (PIVKA-II): 30.25 mAU/mL, HBV DNA < 5× 10^−2^ IU/mL. He was diagnosed with (1) focal liver lesions (hepatic segment of S3 and S4, Child–Pugh grade A) (2); postoperative liver cancer (3); malignant tumor of sigmoid colon (after sigmoid cancer surgery) (4); diabetes mellitus (5); gout; and (6) clonorchiasis sinensis. The patient was discussed by the multidisciplinary team (MDT) of Hepatobiliary Surgery of the First Affiliated Hospital of Guangxi Medical University. The patient’s current hepatic space-occupying lesion was considered to be highly likely to have primary liver cancer, which had unclear boundary and local bile duct compression, and had a history of multiple liver cancer surgical treatment and radical resection of colorectal cancer. In order to clarify the nature of the lesion, it is recommended to improve gastroscopy, HAIC treatment, postoperative adjuvant targeted and immunotherapy, and subsequent evaluation of surgical resection. The MDT team proposed a protocol of arterial infusion chemotherapy combined with sintilimab injection and lenvatinib to transform the tumor and then evaluate surgical treatment. Specific chemotherapy regimen: oxaliplatin 85 mg/m^2^ (2 h), calcium folinate 400 mg/m^2^ (1 h), and rapid infusion of 5-fluorouracil (5-FU) 2,400 mg/m^2^ (46 h). After fully communicating with the patient and his family, the patient chose to undergo surgical resection after conversion therapy and signed a written informed consent for treatment. Then, the first HAIC treatment was performed on 21 March 2022. After the treatment, the patients were treated with renvatinib 8 mg qd + sintilimab 200 mg every 3 weeks, and the next cycle of treatment was performed every 21 days. The second HAIC treatment was the same as the first.

**Figure 2:**
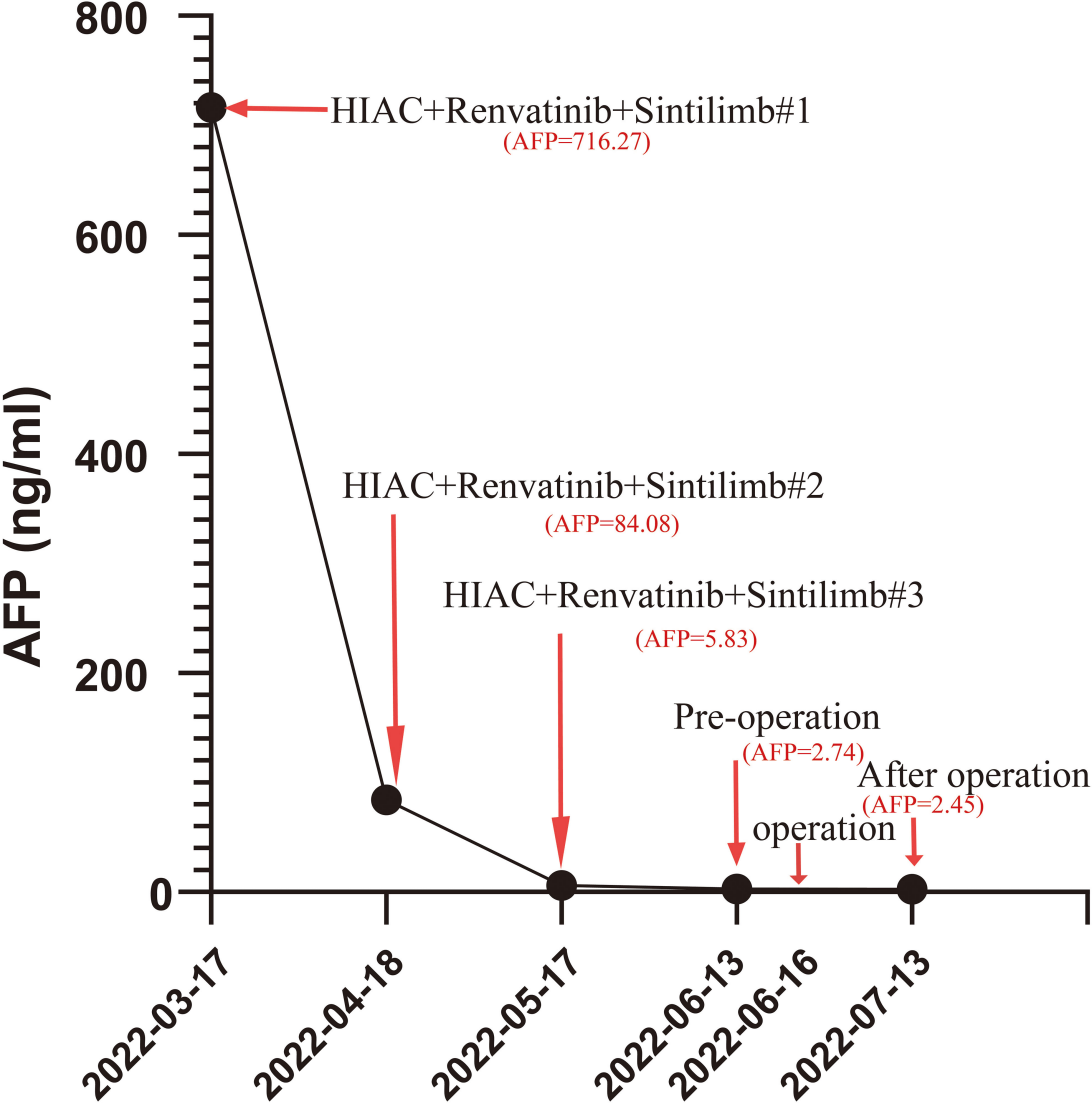
Serum AFP level (ng/mL) decreased significantly during treatment. The number following the pound sign (“#”) represents the cycle of one form of treatment.

**Figure 3:**
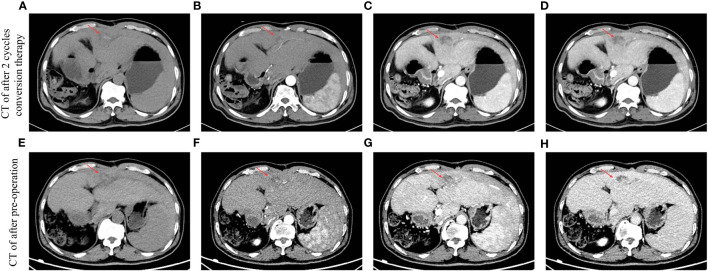
Results of multi-phase CT scans can be evaluated after different periods of conversion therapy. The red arrow indicates an intrahepatic tumor. **(A)** CT image after two cycles of conversion therapy in the period of non-contrast scan. **(B)** CT image after two cycles of conversion therapy in the period of the arterial phase. **(C)** CT image after two cycles of conversion therapy in the period of the portal vein phase. **(D)** CT image after two cycles of conversion therapy in the period of the delay phase. **(E)** CT image after preoperation in the period of non-contrast scan. **(F)** CT image after preoperation in the period of the arterial phase. **(G)** CT image after preoperation in the period of the portal vein phase. **(H)** CT image after preoperation in the period of the delay phase.

**Figure 4:**
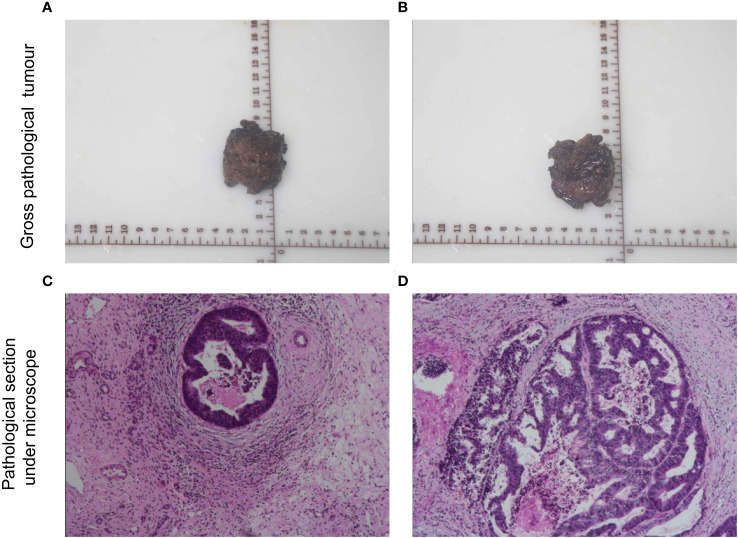
Gross and microscopic pathology of resected specimens. **(A, B)** Gross pathology and **(C, D)** microscope pathology of hematoxylin–eosin staining.

After three cycles of treatment, CT review showed that no significant activity of the intrahepatic S3/4 tumors. Reexamination of serological results suggested AFP is 5.83 ng/mL, PIVKA-II is 35.96 mAU/mL. Child–Pugh grade A and score of 6, indocyanine green (ICG), ICG-R15-minute retention rate of 8.2%. The percentage of the left lateral lobe of the liver in the standard liver volume is 64.5%.. After three cycles HAIC treatments, tumor markers showed a downward trend (**Figure 2**). By using the Modified Evaluation Criteria for Solid Tumor Efficacy (mRECIST) method, the patient’s liver S3 and S4 tumors reached complete response (CR) level, and the tumors showed no significant activity (**Figures 3A–H**). The current treatment reached the expected level, and surgical treatment could be performed. After the contraindication of surgery was excluded, hepatocellular carcinoma resection, intestinal adhesion release, and cholangioplasty were performed on 16 June 2022. The tumor was mainly located in the S3 segment of the liver. The size of the tumor was 2.5×2×1.7 cm^3^. The tumor was irregular in shape, without envelope and borderline clear, the section was yellowish-white, and there was no bleeding and necrosis in the middle. The tumor was close to the intersection of S2 and S3 segments. Because the tumor is close to the liver S2 and S3 segment, the main stem of S2 and S3 segment should be protected and the tumor should be completely resected along the capsule. The residual liver blood supply showed good results after tumor resection. In *in vitro* measurements, the closest distance of the S3 and S4 tumors from the incisal margin was 0 cm (**Figures 4A–D**). Postoperative pathology (left lobe mass of liver): Radical resection specimen after interventional treatment. Most of the tumors were necrotic and the necrosis rate was greater than 95%. Tissue fibrosis, calcification, and more inflammatory cell infiltration. The changes were consistent with interventional therapy. A small amount of live tumor tissue remains (<5%), which is a moderately differentiated adenocarcinoma, and its origin was to be immunohistochemically determined. No satellite nodules, no capsular involvement, no nerve invasion, microvascular invasion (MVI) grade: M0, no tumor involvement at surgical margin, and chronic hepatitis G3S2 changes in the surrounding liver tissue. Special staining results of Ag and PAS supported the above diagnosis. Immunohistochemistry: CK20 (+), CDX-2 (+), CK19 (+), CK7 (−), Hepatocyte (−), Arginase-1 (−), Glypican-3 (−), CD34 (−), HBsAg (−), HBcAg (−), P53 (approximately 90% strong +), P21 (−), NM23 (weak +), VEGF (−), and Ki-67 (approximately 80%+) were considered as the source of gastrointestinal cancer combined with history, morphology, and immunohistochemistry. The patient recovered well after surgery and was discharged 6 days after surgery. The discharge diagnosis was as follows (1): colorectal cancer liver metastases (hepatic segment of S3 and S4, moderately differentiated adenocarcinoma) (2); postoperative liver cancer (3); malignant tumor of sigmoid colon (after sigmoid cancer surgery) (4); diabetes mellitus (5); gout; and (6) clonorchiasis sinensis. There were no significant adverse events during treatment.

## Discussion

Liver metastases detected after radical resection of colorectal cancer are referred to as metachronous liver metastases. Multidisciplinary assistance is recommended for all patients with liver metastases, and the principle of treatment is to pursue a cancer-free state (17). Recent studies have found that the primary colorectal lesion is radical resection without recurrence of the primary lesion, the liver metastases can be completely resected and the amount of liver resection is less than 70% (without cirrhosis), and the liver metastases should be surgically resected (1). Radiofrequency ablation (RFA) of the liver may also be used when the maximum diameter of the metastasis is <3 cm (17) and neoadjuvant therapy may also be considered first. For patients with potentially resectable liver metastases, systemic chemotherapy-targeted immunotherapy after MDT discussion can be evaluated for conversion to resectable liver metastases. In this case, the patient had a history of multiple liver cancer operations and radical resection of colorectal cancer. On admission, AFP, DCP, and CEA were elevated. Considering the complex history of the past, MDT was considered to be more likely to be considered as primary liver cancer due to hepatic space-occupying lesions, unclear lesion boundaries, and local bile duct compression, and radical resection may not be achieved. Resection of the initial liver metastases or ablation of the liver metastases may not be superior to preoperative neoadjuvant therapy. The combination of local HAIC with PD-1 inhibitors and molecularly targeted drugs seems to be a safe and effective conversion approach.

Total neoadjuvant treatment (TNT) (18), which refers to preoperative neoadjuvant chemotherapy and synchronous chemotherapy, can achieve a higher complete response rate, facilitate organ preservation, reduce the occurrence of distant metastasis, and improve long-term survival (19). Conventional systemic chemotherapy for colorectal cancer includes FOLFOX (oxaliplatin + fluorouracil + aldohydrofolic acid), FOLFIRI (irinotecan + fluorouracil + aldohydrofolin), CapeOX (capecitabine + oxaliplatin), or FOLFOXIRI (oxaliplatin + irinotecan + fluorouracil + aldohydrofolin), and other regimens can control tumor growth and prolong overall survival and disease-free survival of patients (20, 21).

Cytotoxic T-lymphocyte associated protein 4 (CTLA4) antibody and programmed cell death 1 (PDCD1, also known as PD-1) antibody are representative drugs of immune checkpoint tumor suppression, which can enhance the activity of immune cells to kill tumor cells, and are widely used in tumor immunotherapy (14, 15). Sintilimab injection is a PD-1 inhibitor. It is widely used in lung cancer, liver cancer, esophageal cancer, and other tumor fields. Multi-target tyrosine kinase inhibitors not only target factors that promote tumor angiogenesis, but also inhibit a variety of kinases related to tumorigenesis. Lenvatinib not only inhibits the kinase activity of VEGFR1-3, but also inhibits FGF and its receptors FGFR1–4, PDGFRα, and stem cell factor receptor, thereby inhibiting tumor blood vessels (16). For example, in the transformation of primary liver cancer, HAIC plus PD-1 combined with tyrosine kinase inhibitor can achieve complete imaging and pathological remission of tumor lesions. HAIC + targeting + immunity can benefit tumor patients for the following reasons (1): Oxaliplatin itself has strong cytotoxic activity, which can kill tumor cells by upregulating the expression of apoptosis-related genes (22), and oxaliplatin can have synergistic effects with a variety of drugs, resulting in it having more powerful anti-tumor effects and less side effects (2). After interventional chemotherapy, the tumor microenvironment changes; liver cancer cells, apoptosis, necrosis, tumor destruction, and a state of hypoxia in the tumor body can enhance the expression activity of vascular endothelial growth factor (VEGF), which will also block the activation of antigen-presenting cells on T lymphocytes. In addition, the necrosis of tumor cells leads to the release of tumor neoantigens, promoting the recruitment and activation of dendritic cells in the microenvironment, changing the tumor microenvironment’s function from immune suppression to immune support, and promoting the effect of immunotherapy. On the other hand, chemotherapy can induce immunogenic death of tumor cells and reduce “non-target” immunosuppression in the tumor microenvironment, thereby improving tumor antigenicity. Therefore, based on the basic treatment of chemotherapy, combined with blocking PD-1/PD-L1 channel and anti-VEGF targeted therapy, the anti-tumor effect can be improved (3). Local perfusion chemotherapy also has a good therapeutic effect on small subfoci, hidden foci, and cancer thrombus invading large blood vessels. In this case, immediate hepatic arterial local infusion chemotherapy + renvastinib + sintilimab was used instead of the conventional systemic intravenous FOLFOX4. After three cycles of treatment, the patient’s imaging findings showed that the tumor did not show significant activity, the A-fetoprotein decreased to normal, the abnormal prothrombin decreased significantly, and the efficacy was evaluated as complete remission (CR). The left external lobe of the liver was excised. Postoperative pathology showed partial remission of left lobe mass (PR), indicating that sequential target immunity of chemotherapy may be an effective conversion therapy for patients with colorectal cancer liver metastasis. In addition, for oncology diseases, the MDT treatment mode is an effective means (23, 24), especially for patients with multiple tumor history, and MDT treatment mode can formulate different treatment goals and give individual treatment plans according to individual conditions of patients. This case demonstrates that the MDT mode plays a positive role in the optimization of the treatment plan for liver metastasis of bowel cancer.

## Conclusion

The treatment process of this case of colorectal cancer with liver metastasis provides a good reference for the study of comprehensive treatment of colorectal cancer with liver metastasis. The multi-disciplinary and multi-method combined treatment mode is an inevitable trend in the treatment of metastatic liver cancer. Our case also provides a new therapeutic strategy for conversion therapy or preoperative neoadjuvant therapy for colorectal cancer with liver metastases. As for the efficacy of the treatment regimen adopted in this study for colorectal cancer liver metastasis, a large number of cases are still needed to be confirmed in the future.

## Data availability statement

The raw data supporting the conclusions of this article will be made available by the authors, without undue reservation.

## Ethics statement

The present study was approved by Medical Ethics Committee of the First Affiliated Hospital of Guangxi Medical University (the approval number is 2023-E637-01). The studies were conducted in accordance with the local legislation and institutional requirements. The participants provided their written informed consent to participate in this study. Written informed consent was obtained from the individual(s) for the publication of any potentially identifiable images or data included in this article.

## Author contributions

KP: Writing – original draft, Data curation, Formal Analysis, Validation, Visualization. YL: Validation, Visualization, Writing – original draft. HS: Writing – review & editing, Project administration, Resources. CL: Validation, Visualization, Writing – original draft. ZH: Formal Analysis, Writing – original draft. YW: Formal Analysis, Writing – original draft. XL: Writing – review & editing, Conceptualization, Methodology. TP: Writing – review & editing, Conceptualization, Resources. MP: Writing – review & editing, Project administration, Resources, Supervision. GZ: Writing – review & editing, Conceptualization, Data curation, Funding acquisition, Project administration, Resources, Supervision.
